# Successful rescue therapy with eculizumab for probable tislelizumab-related MMM overlap syndrome with dual positivity for anti-acetylcholine receptor and anti-titin antibodies: a case report and literature review

**DOI:** 10.3389/fimmu.2026.1873528

**Published:** 2026-06-10

**Authors:** Yi-Xiao Li, Yan-Lei Hao

**Affiliations:** Department of Neurology, Ningbo Taikang Hospital, Ningbo, China

**Keywords:** eculizumab, immune checkpoint inhibitors, MMM overlap syndrome, myasthenia gravis, rescue therapy, tislelizumab

## Abstract

**Background:**

While immune checkpoint inhibitors (ICIs) have revolutionized cancer treatment, they can trigger diverse immune-related adverse events (irAEs). Among these, ICI-related myocarditis, myositis and myasthenia gravis (MMM) overlap syndrome (ICI-MMM) is a rare but potentially fatal complication. Conventional immunotherapy often exhibits limited efficacy against ICI-MMM, which is associated with high mortality rates. Thus, there is an urgent need for novel and effective strategies to mitigate its life-threatening outcomes.

**Method:**

We conducted a retrospective analysis of the successful rescue use of eculizumab in a patient with tislelizumab-related MMM overlap syndrome who tested seropositive for both anti-acetylcholine receptor (AChR) and anti-titin antibodies. We also performed a focused systematic literature review on the use of complement inhibitor therapy for ICI-related myasthenia gravis and its overlap syndrome.

**Result:**

A 64-year-old male developed ptosis and tetraparesis two weeks following the second infusion of tislelizumab for lung adenocarcinoma. Serological testing revealed dual positivity for anti-AChR antibody and anti-titin antibody. Tislelizumab was immediately withdrawn, and the patient was treated with corticosteroids and intravenous immunoglobulin as first-line therapy. However, his clinical condition deteriorated rapidly, and new symptoms emerged, including chest pain, muscle pain, dysphagia, slurred speech, and dyspnea. Although the absence of histopathological confirmation for myocarditis and myositis, the clinical, laboratory, electrophysiological, and cardiac imaging findings supported the diagnosis of probable ICI-MMM. Rescue therapy with eculizumab was commenced (900 mg weekly for four doses), eliciting rapid and marked clinical improvement. The patient ultimately achieved minimal symptom expression without any exacerbation.

**Conclusion:**

This is the first reported case of successful eculizumab rescue treatment for probable tislelizumab-related MMM overlap syndrome with dual seropositivity. Our finding suggests eculizumab may represent a promising rescue option for ICI-MMM warranting prospective evaluation.

## Introduction

Immune checkpoint inhibitors (ICIs) have transformed the therapeutic landscape of oncology. These monoclonal antibodies target programmed death-1/programmed death ligand-1 (PD-1/PD-L1) and cytotoxic T lymphocyte antigen-4 (CTLA-4) pathways (1). By blocking these inhibitory checkpoints, ICIs enhance anti-tumor immunity but may also trigger immune-related adverse events (irAEs) (2). The concurrent occurrence of multiple irAEs is common, with incidence rates of 10–20% for monotherapy and up to 30–40% for combination regimes of ICIs (3, 4). Myocarditis, myositis, and myasthenia gravis (MMM) overlap syndrome is a rare complication, observed in approximately 0.1–0.3% of patients receiving ICI therapy. Despite its low incidence, it is an extremely severe condition associated with in-hospital mortality rates as high as 60% (5). Early recognition and treatment of ICI-related MMM overlap syndrome (ICI-MMM) lead to improved outcomes (6), underscoring the critical importance of timely diagnosis and aggressive intervention to mitigate its potentially fatal consequences.

The optimal management of ICI-MMM remains uncertain. Conventional treatments, including corticosteroids, intravenous immunoglobulin (IVIG), and plasma exchange (PLEX), often proved unsatisfactory in this syndrome (7). Although monoclonal antibodies such as tocilizumab and rituximab have been used in some severe or refractory cases (5), more effective and safer therapies for ICI-MMM are urgently needed.

Eculizumab is a monoclonal antibody targeting the C5 component of the complement system, thereby inhibiting the formation of the membrane attack complex (MAC) (8). It has demonstrated high efficacy in improving clinical symptoms, reducing exacerbation rates, and achieving minimal symptom expression in myasthenia gravis (MG) (9). Recently, Nelke et al. reported a severe case of pembrolizumab-related MMM overlap syndrome in which muscle biopsy identified substantial complement deposition, providing a mechanistic rationale for the use of eculizumab as rescue therapy (10). Herein, we present a case of lung cancer in which the patient developed probable ICI-MMM with dual seropositivity for anti-acetylcholine receptor (AChR) and anti-titin antibodies after tislelizumab therapy. The patient ultimately achieved minimal symptom expression after receiving eculizumab as rescue therapy. This case suggests that eculizumab, owing to its rapid onset of action, may present a safe and effective rescue option for patients with ICI-MMM.

## Case presentation

A 64-year-old man presented on July 12, 2025, with a four-day history of progressive ptosis and generalized weakness that had developed following his second infusion of tislelizumab. His symptoms had worsened significantly two days prior to admission, manifesting as profound fatigue after minimal activity and weakness involving all four limbs. The patient had been diagnosed with lung adenocarcinoma on May 20, 2025, and received combination therapy (tislelizumab, carboplatin, and pemetrexed) on June 5, 2025, and June 26, 2025, respectively. His past medical history included cerebral infarction, hypertension, pulmonary tuberculosis, and coronary heart disease; he had undergone coronary artery stenting in 2021 with uneventful recovery.

On admission, physical examination showed a cachectic man (weight 49 kg, height 170 cm). Cognition was intact. Neurological examination identified dysarthria, bilateral ptosis, diplopia, and mild weakness of both neck flexors and extensors. Muscle strength [Medical Research Council (MRC) scale] was 4/5 in neck flexors and extensors, 3/5 in the deltoids, biceps, and triceps, and 3/5 in the quadriceps and iliopsoas. Eyelid fatigability testing was positive. Deep tendon reflexes were normal in the lower limbs, and bilateral Babinski signs were absent. No sensory deficits were detected.

Repetitive nerve stimulation testing demonstrated a significant decremental response at low-rate stimulation. Initial laboratory studies, including lactate, procalcitonin, interleukin-6, C-reactive protein, creatine kinase (CK), lactate dehydrogenase, complete blood count, coagulation profile, cardiac troponin T (cTnT), and pro-BNP were all within normal limits. Electrocardiography (ECG) showed sinus tachycardia. Echocardiography was unremarkable. Mediastinal/chest computed tomography (CT) showed no evidence of thymoma. Serological testing by enzyme-linked immunosorbent assay (ELISA) revealed positivity for AChR antibody (7.625 nmol/L; normal range < 0.45 nmol/L) and anti-titin antibody (2.53 nmol/L; normal range < 0.472 nmol/L). Tests for anti-MuSK, anti-LRP4, and serum onconeural antibodies were negative. Based on these findings, a diagnosis of tislelizumab-related MG with positivity for both AChR and anti-titin antibodies was established (MGFA [Myasthenia Gravis Foundation of America] class IIIA). The Quantitative Myasthenia Gravis (QMG) score was 22, and the MG-Activities of Daily Living (MG-ADL) score was 10.

ICI therapy was discontinued immediately. First-line treatment with oral prednisone (1 mg/kg/day) and IVIG (0.4/kg/day for 5 days) was initiated. Despite two weeks of standard treatment, new symptoms emerged, including fever, chest pain, muscle pain, dysphagia, slurred speech, and dyspnea (QMG score 30, MG-ADL score 18). A preliminary diagnosis of myasthenic crisis (MC) was considered. He received orotracheal intubation and invasive mechanical ventilation with conventional volume-controlled ventilation mode.

**Figure 1:**
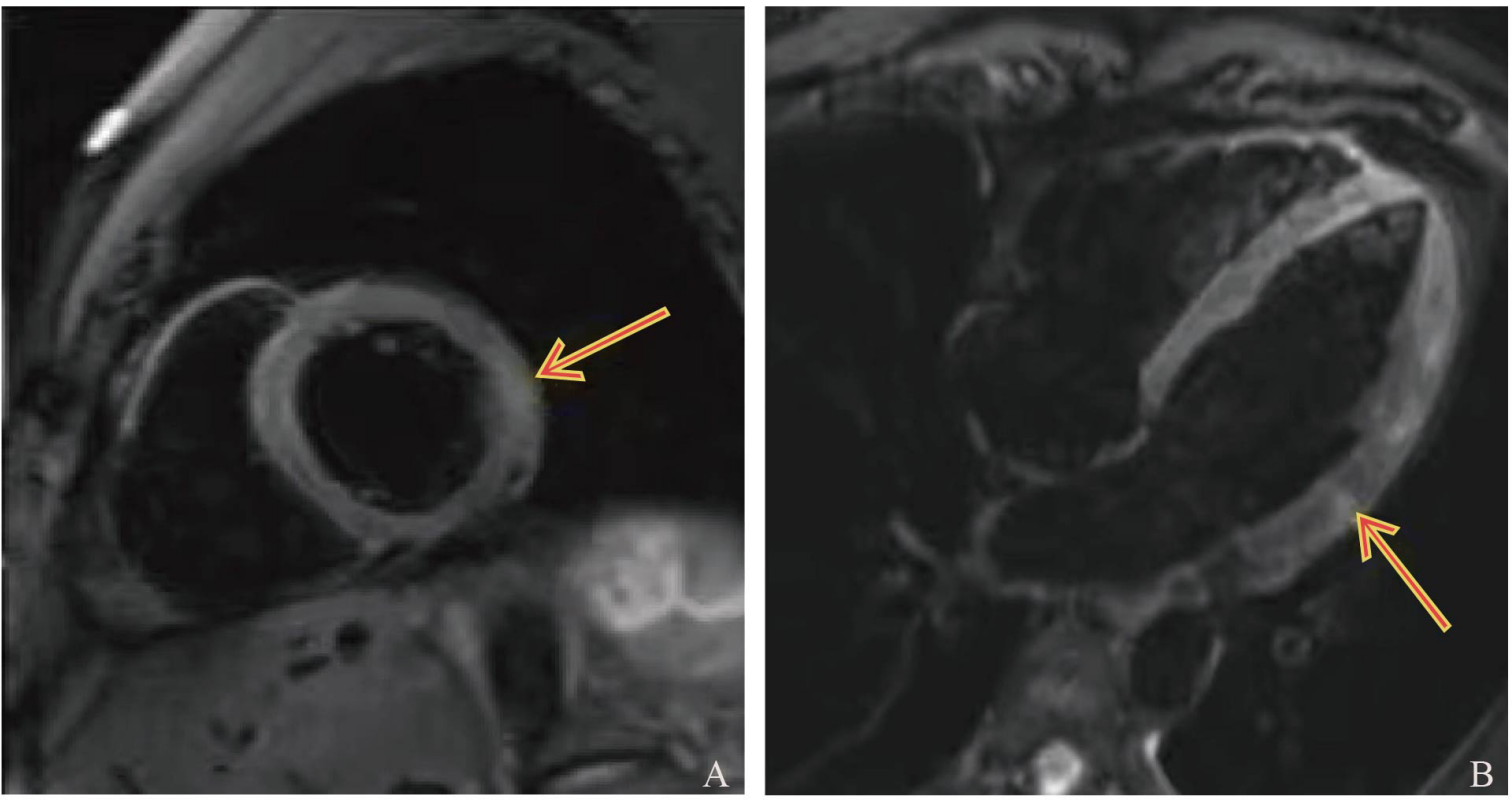
CMR imaging findings of acute myocarditis. **(A)** Short-axis T2-STIR images revealed segmental hyperintensity in the left ventricular lateral free wall (arrow), consistent with acute interstitial myocardial edema. **(B)** Long-axis LGE images revealed patchy and linear hyperenhancement (arrow) in the inferolateral wall of the left ventricle, predominately involving the mid-myocardial and subepicardial layers. CMR, cardiac magnetic resonance; LGE, late gadolinium enhancement; T2-STIR, T2-weighted Short Tau Inversion Recovery.

Further diagnostic workup revealed respiratory acidosis (pH 7.32, pCO_2_ 57 mmHg), elevated CK level of 498 U/L (3× ULN) and markedly increased cTnT level of 1248 ng/L (78× ULN). Chest CT confirmed right lobar pneumonia. ECG showed a first-degree atrioventricular block. Nerve conduction studies demonstrated normal findings in bilateral median, ulnar, tibial, and peroneal motor nerves, as well as bilateral median, ulnar, and sural sensory nerves, ruling out peripheral neuropathy. Needle electromyography (EMG) revealed abundant fibrillation potentials in the bilateral biceps brachii muscles, indicative of ongoing muscle fiber necrosis. In the left deltoid, biceps brachii, tibialis anterior, and iliopsoas muscles, voluntary contraction elicited low-amplitude, short-duration, polyphasic motor unit action potentials accompanied by early recruitment. Collectively, these electrophysiological features are highly consistent with widespread, active myofiber damage characteristic of an inflammatory myopathy. Repetitive nerve stimulation continued to a pathological decremental response. Echocardiography demonstrated a non-dilated left ventricle with preserved ejection fraction. Cardiac magnetic resonance imaging (MRI) detected interstitial edema in the left ventricular wall, suggestive of acute myocarditis (**Figure 1**).

Given the high suspicion of tislelizumab-related MMM overlap syndrome, PLEX was considered but was not available at our center. After multidisciplinary discussion and informed consent from the family, we initiated rescue therapy with the complement C5 inhibitor eculizumab. Treatment followed the standard regimen for refractory generalized MG: 900 mg intravenously weekly for four doses, then 1200 mg every two weeks. Owing to the patient’s critical clinical status and uregnt need for salvage therapy, meningococcal vaccination could not be administered at least 2 weeks in advance as routinely recommended; instead, vaccination was given concurrently with eculizumab initiation. To reduce the risk of encapsulated bacterial infections, antibiotic prophylaxis with benzathine penicillin was administered concomitantly and continued for additional 10 days.

The patient showed a rapid clinical response following eculizumab initiation. By day 3 after the first infusion, he exhibited ECG normalization, and improvement in CK (from 498 U/L to 172 U/L) and cTnT (from 1248 ng/L to 760 ng/L) levels. By day 6 after the second infusion, both CK and cTnT levels had returned to normal. His symptoms improved remarkably, allowing successfully liberation from mechanical ventilation on day 7 post the second infusion, followed by independent ambulation with a walker (QMG score 17, MG-ADL score 11). By day 7 following the third infusion, limb muscle weakness further improved (QMG score 13, MG-ADL score 8). His nasogastric tube was removed, and the patient resumed oral intake. Eight days after completion of the four doses, he regained near-full independence in activities of daily living (QMG score 6, MG-ADL score 4) and was discharged.

**Figure 2:**
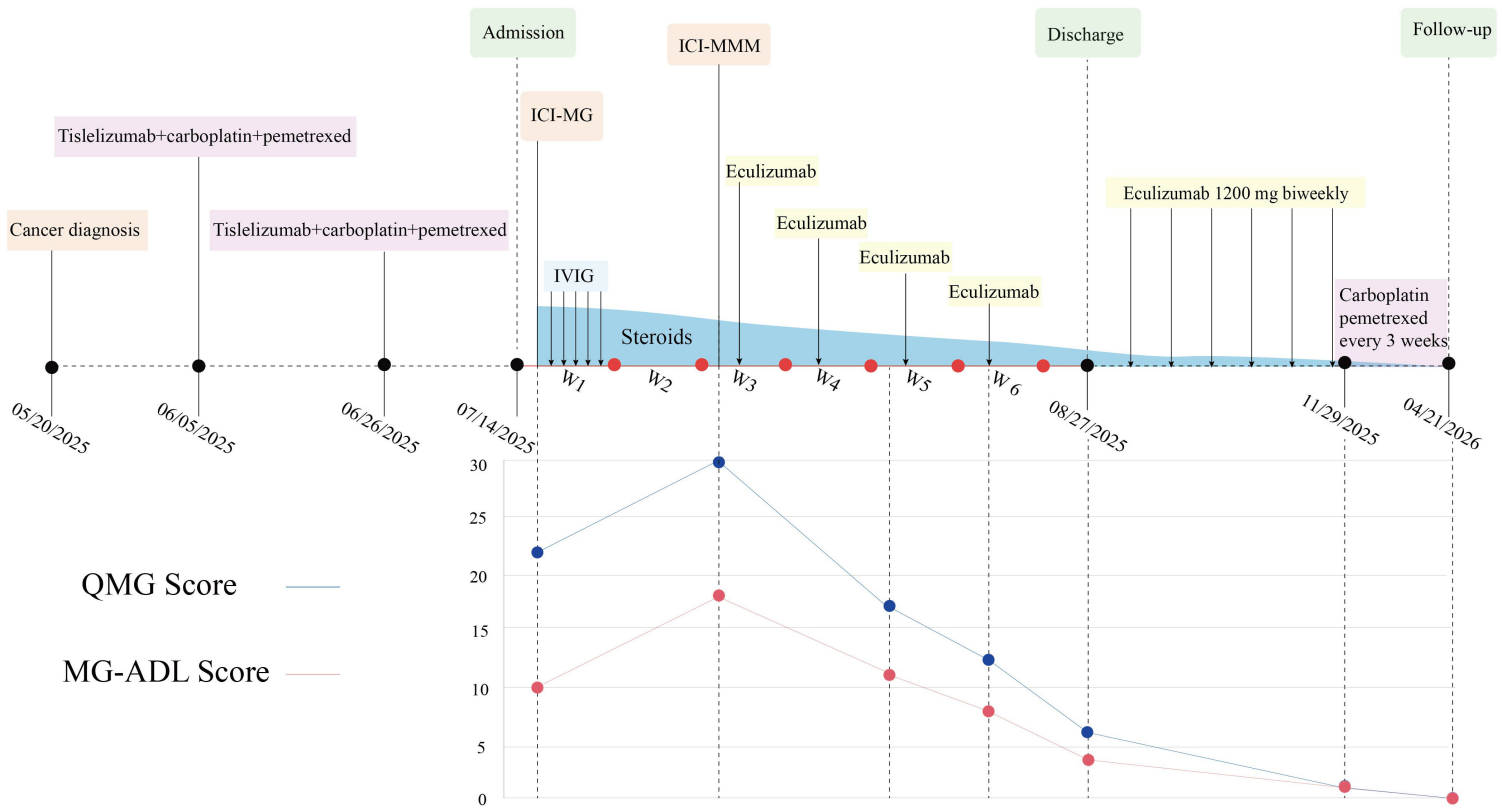
Clinical findings and change of the QMG and MG-ADL scores during the course of disease. ICI-MG, immune checkpoint inhibitor-related myasthenia gravis; ICI-MMM, immune checkpoint inhibitor-related myocarditis, myositis and myasthenia gravis overlap syndrome; IVIG, intravenous immunoglobulin; MC, myasthenic crisis; MG, myasthenia gravis; MG-ADL, myasthenia gravis-Activity of Daily Living; QMG, Quantitative myasthenia gravis; W, week.

During the three-month follow-up period, he continued biweekly maintenance eculizumab infusion (1200 mg) in the outpatient clinic. Prednisone was gradually tapered to a maintenance dose of 5 mg daily during eculizumab treatment. He achieved minimal symptom expression (QMG score 1, MG-ADL score 1) and experienced no exacerbation. During the four-month follow-up, cardiac MRI revealed complete resolution of myocardial late gadolinium enhancement. A single follow-up ELISA performed at this time point confirmed seronegativity. At the eight-month follow-up, QMG and ADL remained stable (QMG score 0, MG-ADL score 0). The patient’s primary tumor remained radiologically stable under maintenance chemotherapy with capecitabine and pemetrexed, without evidence of disease progression. ICI was not rechallenged. Long-term clinical and laboratory monitoring is ongoing. **Figure 2** shows the key clinical findings, disease course, and serial changes in QMG and MG-ADL scores following immunotherapy (**Supplementary Table 1**).

## Literature review

A comprehensive literature search was performed using MEDLINE/PubMed with the terms: “Myasthenia gravis,” “Myocarditis,” “Myositis,” combined with “Immune checkpoint inhibitors,” “Programmed Cell Death 1,” “PD-1,” “PD-L1,” “Cytotoxic T lymphocyte antigen-4,” “CTLA-4,” “Pembrolizumab,” “Nivolumab,” “Ipilimumab” “Ravulizumab,” “Zilucoplan,” or “Tislelizumab,” as well as “Eculizumab” or “Complement inhibitors.” The search was limited to English-language articles published between January 1986 and April 2026. Finally, 289 publications were initially identified. After removing 101 duplicates, 188 articles underwent title and abstract screening, of which 97 were excluded (59 irrelevant to the research topic, 28 failing to meet inclusion criteria, 10 *in vitro* studies). The remaining 91 articles underwent full-text assessment, and 87 articles were further excluded due to ineligibility (44 irrelevant to topic, 42 with insufficient patient clinical data, 1 duplicate publication). Finally a total of four articles encompassing five reported cases were included in the review (**Table 1**) (8, 10–12).

**Table 1:** Review of cases on complement inhibitor therapy for ICI-related MG and its overlap syndrome.

Author/year	Age/gender	Mechanisms/type of ICIs	Cancers	ICI cycle	History of MG before ICIs	Clinical symptoms	Management	Outcomes	When to restart ICI therapy
Nelke et al/2024 (10)	47/F	PD-1/pembrolizumab	Breast cancer	1	AChR-positive MG with thymoma removal	Progressive muscle weakness and difficulties in swallowing and speaking; myositis-myocarditis; MC overlap; MC	Steroids, immunoadsorption, eculizumab	Good; MG responded to eculizumab	Without rechallenging ICI
Zadeh et al/2024 (11)	80/M	PD-1/pembrolizumab	Squamous cell carcinoma of bladder	1	No history of MG	AChR-positive MG, myositis, and myocarditis, impending MC	Steroids, eculizumab, ravulizumab	Poor; MG responded to eculizumab; died of metastatic cancer 8 months later	Terminated
Zadeh et al/2024 (11)	58/M	PD-1/pembrolizumab	Merkel cell cancer	No irAEs for 22 months	Refractory thymoma associated AChR-positive MG	Mild, indolent retinal vasculitis	Steroids, eculizumab	Good; MG remained stable on eculizumab.	Discontinuation of pembrolizumab for 5 months resulted in cancer recurrence, pembrolizumab was resumed with peri-infusion pulse prednisone
Fionda et al/2024 (8)	76/M	PD-1/pembrolizumab	Metastatic colorectal cancer	2	Non-thymomatous generalized AChR-positive MG	Myasthenic exacerbation, dysarthria, dysphagia, dyspnea, and bilateral ptosis	Steroids, IVIG, eculizumab	Good; MG remained stable on eculizumab	Pembrolizumab regularly continued, with a good control of cancer progression
Huang et al/2025 (12)	75/M	PD-1/pembrolizumab	Cholangiocarcinoma	1	No history of MG	Weakness in all limbs, fatigue and rapidly progressing to an impending MC	Steroids, eculizumab	Good; MG remained stable on eculizumab	Without rechallenging ICI
Present case	64/M	PD-1/tislelizumab	Lung cancer	2	No history of MG	Progressive ptosis and generalized weakness; MC	Steroids, IVIG, and eculizumab	Good; MG remained stable on eculizumab	Without rechallenging ICI

AChR, acetylcholine receptor; F, female; ICI, immune checkpoint inhibitor; irAE, immune-related adverse event; IVIG, intravenous immunoglobulin; M, male; MC, myasthenic crisis; MG, myasthenia gravis; PD-1, programmed cell death receptor 1.

## Discussion

Although ICI-related irAEs are rare, they can be serious and life-threatening, particularly when they involve concomitant myositis, myocarditis, and MG (5). MMM overlap syndrome typically occurs within the first month after ICI treatment, is more common in patients treated with PD-1 inhibitors, and has a median age of onset of approximately 70 years (5). To date, no case of tislelizumab-related MMM has been previously documented. In total, six cases (including ours) of complement inhibitor therapy for ICI-related MG (ICI-MG) and overlap syndrome (with myocarditis and/or myositis) have been reported (**Table 1**). The most commonly reported signs and symptoms include diplopia, muscle weakness, myalgias, dyspnea, and dysphagia. All patients with ICI-MG and overlap syndrome receiving eculizumab to date have tested positive for anti-AChR. Our patient was additionally seropositive for anti-titin antibody, a subtype of antistriated muscle antibodies. To our knowledge, this is the first reported case of probable tislelizumab-related MMM with dual anti-AChR and anti-titin antibody positivity that responded rapidly to eculizumab rescue therapy.

Each of the three components should be considered separately in the differential diagnosis of ICI-MMM. For ICI-related myositis, key differential diagnosis includes fasciitis, polymyalgia rheumatica, and neuromyopathy (13). Clinical features, EMG examination, and CK elevation facilitate the diagnosis of immune-mediated myositis. For immune-mediated myocarditis, differential diagnosis includes heart failure, acute myocardial infarction, atrial fibrillation, viral myocarditis, and Takotsubo cardiomyopathy (14). Cardiac MRI is the preferred imaging modality, and endomyocardial biopsy is the gold standard for myocarditis (15). Due to concerns about potential risks and complications associated with endomyocardial and muscle biopsies, the patient’s family declined all these diagnostic invasive procedures. Despite the lack of histopathological confirmation, cardiac MRI findings provided supportive evidence for a diagnosis of myocarditis. Based on the International Cardio-Oncology Society consensus statement (16), our patient fulfilled the diagnostic criteria of probable ICI-related myocarditis. Regarding neuromuscular junction disorders, Lambert-Eaton myasthenic syndrome (LEMS) associated with ICIs is a primary differential diagnosis for ICI-MG due to their clinical similarities. LEMS is typically mediated by antibodies against voltage-gated calcium channels, predominately affects proximal muscles, shows improvement with repetitive muscle contraction, and exhibits an incremental response on repetitive nerve stimulation studies. Currently, there are no established diagnostic criteria for ICI-MG, but the presence of anti-AChR and anti-titin antibodies in our case strongly supports this diagnosis. Diagnosing ICI-MG can sometimes be challenging, as anti-AChR antibodies are positive in less than 60% of affected patients, and myositis-related symptoms may mimic myasthenia (17). In combination with antibody tests, EMG findings and clinical features, the diagnosis of ICI-MG was highly consistent with our patient. Although the clinical, laboratory, electrophysiological, and cardiac imaging findings were highly consistent with myocarditis, myositis, and MG overlap syndrome, the absence of histopathological confirmation limits definitive conclusions regarding the underlying pathological mechanisms, particularly complement deposition and immune cell infiltration in affected tissues, and our observations regarding the efficacy of eculizumab should be considered preliminary until supported by further pathological and mechanistic studies.

Unlike classical MG, ICI-MG frequently presents with more severe weakness, bulbar and respiratory involvement, and rapid progression to MC (18). It has been observed that 30–40% of ICI-MG cases overlap with myositis, and 8–39.7% with myocarditis (5, 19). ICI-MMM significantly worsens the prognosis, with mortality rates ranging from 40% to 60% (5, 19, 20). These findings underscore the urgency of early recognition and effective management of ICI-MMM. Given the frequent overlap with myositis and myocarditis, the diagnosis of any one of these three conditions should prompt comprehensive investigation for the other two. Routine assessment of CK and cTnT levels during the initial cycles of treatment may be warranted.

In anti-PD-1-associated MG, anti-AChR antibody positivity ranges widely from 20% to 73% (21), while anti-titin antibodies are seldom reported (22). Our case represents the first documented instance of dual anti-AChR and anti-titin antibody positivity in PD-1 inhibitor-related MMM. Anti-titin antibody is often linked with thymoma and myocarditis (23). Though no evidence of thymoma was found, this still raises a critical question regarding the conceptual boundary between ICI-MG and classical MG. It remains challenging to definitively distinguish whether this case represents a *de novo* neurological irAE directly induced by tislelizumab, or a pre-existing subclinical MG that was unmasked or accelerated by ICI-induced immune activation. Patients with pre-existing autoantibodies may increase the risk of neurological irAEs (24). Prospective studies are warranted to monitor the presence and titers of MG-associated antibodies before, during, and after ICI treatment, and to correlate these dynamics with the development of neuromuscular syndromes.

Given the proposed pathogenic role of anti-AChR antibody and anti-titin antibodies in ICI-MMM, early immunotherapy may offer a critical treatable window for disease control (25). Given the rarity of ICI-MMM, evidence-based treatment protocols are lacking. Current management strategies are largely extrapolated from those used for classical MG or myositis, with immunosuppression—such as corticosteroids, IVIG, PLEX—serving as the mainstay of therapy. However, escalation to more potent immunosuppressive drugs such as rituximab and tocilizumab has also been reported in refractory cases (5, 26). These therapeutic options further reflect the complexity of managing ICI-MMM. Early immunomodulation with IVIG could prevent the potential initial worsening of ICI-MG secondary to corticosteroids, supporting its use from the outset alongside these agents (27). However, as observed in our patient, early administration of steroids and IVIG failed to halt rapid progression to respiratory failure requiring intubation and mechanical ventilation, necessitating a more aggressive approach. Anti-titin antibody is generally associated with more severe disease and higher hospitalization rates (28). Consistent with this treatment-refractory nature, our patient showed a poor response to combined therapies including corticosteroids and IVIG, and progressed to MC. This case highlights the need for clinicians to maintain a high index of suspicion for refractory ICI-MG, particularly when anti-titin antibody is present. Aggressive monitoring and early escalation of therapy are warranted to alleviate symptoms and reduce mortality.

PLEX was unavailable at our center; consequently, eculizumab was selected as an alternative. Eculizumab, a monoclonal antibody targeting complement C5, inhibits the cleavage of C5 into C5a and C5b within the complement activation cascade, thereby preventing the formation of the C5b-induced MAC (29). It is approved for the treatment of patients with refractory AChR antibody-positive generalized MG (9). Complement activation has been identified as playing an important role in the pathogenesis of ICI-MMM, justifying the use of targeted drugs such as eculizumab (10). Several case reports have documented the tentative use of eculizumab in ICI-MG and ICI-MMM, as detailed in **Table 1**. These findings suggest that eculizumab, when used as an add-on therapy to steroids, can induce rapid and significant clinical improvement with a favorable safety profile. Notably, while the rapid clinical improvement following eculizumab administration is compelling, we cannot entirely exclude the possibility of a delayed therapeutic effect from the preceding high-dose methylprednisolone and IVIG, which may have created a more favorable immunological milieu for subsequent C5 inhibitor. Meanwhile, specific regimens (including dosage per administration and treatment duration) require further investigation.

Another challenge in the management of ICI-MMM is ICI rechallenge. Tumor progression has been reported as the main cause of death in patients who experience irAEs; yet alternative treatment options are limited. An anecdotal case described a patient with ICI-MG who discontinued pembrolizumab and achieved clinical improvement with eculizumab; however, cancer recurred 5 months after ICI discontinuation, leading to cautious re-initiation of ICI therapy (8). The safety of restarting ICIs after the resolution of severe irAEs remains poorly characterized. Inan et al. resumed nivolumab monotherapy 1 month after clinical stabilization of a patient with ICI-MMM (30). The patient remained neurologically stable for 4 months without any recurrent irAEs. In the present case, ICI rechallenge was not pursued, and the patient was instead maintained on capecitabine and pemetrexed. The decision was guided by the patient’s severe grade ≥ 3 neurological irAE, his favorable oncologic stability under alternative systemic therapy, and alignment with the clinical practice guidelines from the Society for Immunotherapy of Cancer (SITC) (31). In the future similar cases, decisions regarding ICI rechallenge should emerge from careful multidiscipline risk-benefit assessment, taking into account the completeness of neurological recovery, the availability of other effective oncologic therapies, the pace and status of tumor progression, and shared decision-making with the patient and caregivers. Close, ongoing monitoring of both neurological and oncologic outcomes remains essential in any scenario where ICI rechallenge is considered.

## Limitations and future directions

Several limitations need to be considered when interpreting our finding. Our case is anecdotal, which limits generalizability. Moreover, we reviewed only a limited number of previous case reports on C5 complement inhibitors for ICI-MG and ICI-MMM, which may introduce publication bias. In addition, the potential underreporting may hinder the establishment of a causal relationship between ICI treatment and the MMM overlap syndrome, affecting our ability to make strong recommendations regarding management. To advance clinical investigation, several steps are needed. First, systematic case collection through international registries (e.g., neurological irAE or MGFA databases) would clarify the incidence, spectrum, and outcomes of ICI-MMM. Second, prospective studies are required to validate complement inhibitor therapy in refractory ICI-MMM. Third, multicenter case series should establish standardized protocols comparing conventional (corticosteroids, IVIG, PLEX) versus novel (efgartigimod, complement inhibitors) immunotherapies. Finally, rigorous immunohistochemical and serological investigations are warranted to confirm antibody expression in tumor tissues and elucidate ICI-triggered autoimmune mechanisms.

## Conclusion

We herein report a patient with lung adenocarcinoma who developed probable MMM with concurrent anti-AChR and anti-titin antibody positivity following treatment with tislelizumab. Even if histopathological confirmation of myocarditis, myositis, and complement-mediated tissue injury, was not available in this patient, successful rescue treatment with eculizumab was achieved. The observations suggest that eculizumab may serve as a promising therapeutic alternative for ICI-MMM. Early recognition and immediate initiation of appropriate treatment are critical for improving the prognosis in patient with ICI-MMM. Further prospective studies and pooled analyses are warranted to clarify the optimal timing, dosing, safety, and efficacy of complement inhibition in ICI-MMM.

## Data Availability

The original contributions presented in the study are included in the article/**Supplementary Material**. Further inquiries can be directed to the corresponding author.
