# Supplementary material for: Successful rescue therapy with eculizumab for probable tislelizumab-related MMM overlap syndrome with dual positivity for anti-acetylcholine receptor and anti-titin antibodies: a case report and literature review
Source: Front Immunol. 2026 Jun 10;17:1873528. doi: 10.3389/fimmu.2026.1873528 (PMC13290910; doi:10.3389/fimmu.2026.1873528)
Supplement: Supplementary file 1 [file Table1.docx]

**Supplementary table 1 Clinical scales scores during evaluation period**

| **MG-ADL** | **Baseline** | **2W** | **4W** | **5W** | **6W** | **18W** | **38W** |
| --- | --- | --- | --- | --- | --- | --- | --- |
| Talking | 1 | 2 | 1 | 1 | 1 | 0 | 0 |
| Chewing | 1 | 2 | 2 | 1 | 1 | 0 | 0 |
| Swallowing | 1 | 3 | 2 | 1 | 0 | 0 | 0 |
| Breathing | 1 | 3 | 1 | 1 | 0 | 0 | 0 |
| Brushing teeth or combing hair | 1 | 2 | 2 | 1 | 0 | 0 | 0 |
| Arising from a chair | 1 | 2 | 1 | 1 | 0 | 0 | 0 |
| Double vision | 2 | 2 | 1 | 1 | 1 | 0 | 0 |
| Eyelid drop | 2 | 2 | 1 | 1 | 1 | 1 | 0 |
| *Total* | 10 | 18 | 11 | 8 | 4 | 1 | 0 |
| **QMG** |  |  |  |  |  |  |  |
| Double vision on lateral gaze | 1 | 1 | 1 | 1 | 1 | 0 | 0 |
| Ptosis (upward gaze) | 1 | 2 | 1 | 1 | 1 | 1 | 0 |
| Facial muscles | 0 | 1 | 2 | 1 | 0 | 0 | 0 |
| Swallowing 4 oz water | 1 | 2 | 2 | 1 | 0 | 0 | 0 |
| Speech after counting aloud | 1 | 2 | 1 | 1 | 0 | 0 | 0 |
| Right arm outstretched | 2 | 3 | 1 | 1 | 0 | 0 | 0 |
| Left arm outstretched | 2 | 3 | 1 | 1 | 0 | 0 | 0 |
| Forced vital capacity | 1 | 1 | 1 | 1 | 1 | 0 | 0 |
| Rt-hand grip, kg | 3 | 3 | 1 | 1 | 1 | 0 | 0 |
| Lt-hand grip, kg | 3 | 3 | 1 | 1 | 1 | 0 | 0 |
| Head lifted | 3 | 3 | 1 | 1 | 0 | 0 | 0 |
| Right leg outstretched | 2 | 3 | 2 | 1 | 1 | 0 | 0 |
| Left leg outstretched | 2 | 3 | 2 | 1 | 0 | 0 | 0 |
| *Total* | 22 | 30 | 17 | 13 | 6 | 1 | 0 |

**Abbreviations:** MG-ADL, myasthenia gravis-Activity of Daily Living; QMG, Quantitative myasthenia gravis.
